# What are the effects of supporting early parenting by enhancing parents’ understanding of the infant? Study protocol for a cluster-randomized community-based trial of the Newborn Behavioral Observation (NBO) method

**DOI:** 10.1186/s12889-018-5747-4

**Published:** 2018-07-04

**Authors:** Ingeborg Hedegaard Kristensen, Hanne Kronborg

**Affiliations:** Section of Nursing, Department of Public Health, Aarhus University, Bartholins Allé 2, 8000 Aarhus C, Denmark

**Keywords:** Parenting programme, Newborn behavioral observation, Early intervention, Universal intervention, Health visitors, Family relationship, Community setting

## Abstract

**Background:**

Support to strengthen the early parent-infant relationship is recommended to ensure the infant’s future health and development. Little is known about the universal approaches taken by health visitor to support this early relationship. The aim of this study is to investigate the effects of health visitors’ use of the Newborn Behavioral Observation (NBO) method among new parents.

**Methods:**

This is a cluster-randomised community-based study implemented in four Danish municipalities. Health visitors will conduct the trial, and the geographical districts they work in will constitute the clusters as units of randomisation. The participants will be approximately 2800 new families, randomised into an intervention or a comparison group according to their health visitor. The families are recruited at the first postpartum home visit. Parents in both groups receive care as usual: parents in the intervention group also receive the standardised NBO method in home visits performed from 3 weeks to 3 months postpartum. Data consist of self-reported parent questionnaires and video recordings of a selected group of vulnerable first-time mothers recorded 4 months postpartum. The self-reported data are obtained: at baseline 1 week postpartum and then at follow-up 3, 9 and 18 months postpartum. Data will be analysed using the intention-to-treat method and the analyses will include comparison of change in the primary variables across time supplemented by multiple regression analysis. The primary study outcomes are measured by the following factors: parental confidence, infants’ socio-emotional development and mother-infant relationship. Other measures include parental mood and stress, breastfeeding duration and utility of the health visitor services. Data collection among the health visitors in both groups will serve to monitor any change in practice regarding the work with early parent-infant interactions.

**Discussion:**

This protocol describes an evaluation of the NBO method used universally in health visiting practice. The intervention seeks to support early parenting by increasing parents’ understanding of their infants’ cues. The NBO is currently implemented in Denmark even though an evaluation of the NBO has yet to be made in a community setting in Denmark and internationally. The study may contribute to building an increasingly evidence-based practice for health visitors.

**Trial registration:**

ClinicalTrials.gov ID: NCT03070652. Registered February 22, 2017.

## Background

### Foundation of early parenting and parents at risk

The postnatal period is important for establishment of a healthy parent-infant relationship. The quality of this relationship is associated with the infant’s subsequent physical [1, 2] and psychosocial health [3–6] and cognitive development [7, 8].

In a general population of parents, a number of factors influencing the early parent-infant relationship have been identified [9–11]. First-time parents, in particular, tend to experience insecurity in responding to their infant’s cues [12], and fathers express more insecurity than mothers [13]. Insecurity is often present in the first weeks postpartum and among parents experiencing that their infant cries persistently or has sleep disturbances [14–17]. More than half of first-time mothers have reported that they experience difficulties and need of support in the early postpartum period [18, 19].

Preterm birth is another factor that may disturb the early parent-infant relationship. Parents of premature infants often experience anxiety, depression and decreased confidence in their own parenting capabilities. Combined with sorrow, stress, uncertainty and helplessness caused by the infant’s condition, this may impair parent-infant interaction [20, 21]. Worldwide, preterm birth is experienced by 11% of mothers; in developed countries by 6–8% [22].

Depressive symptoms in the postpartum period are also likely to impact on the quality of the parent-infant relationship [23–25]. Treatment of the parents’ depressive symptoms alone may be insufficient to protect children from poor long-term outcomes [23]. The estimated prevalence of parental postpartum depression is approximately 7–14% [25–27]. In previous research, one in five new Danish families showed signs of inadequate parenting due to low parenting confidence or parents had symptoms of depression [28].

Relationship disturbances are often more profound in families with cases of abuse, critical illness, domestic conflicts or mental illness, and may result in parent-infant-relationship disorder [9–11, 29].

### Effect of universal parenting support on the quality of the early parent-infant relationship remains unknown

The WHO and the Danish National Health Promoting Guidelines recommend early universal parental support [30, 31]. However, the effects of universal strategies for supporting early parenting remain unknown. The few reliable intervention studies available have focused mainly on parents at risk, such as mothers living in disadvantaged settings (32) or young less-educated mothers (33). These studies have shown that intervention brings significant long-term improvements in mortality for both mothers and infants [32] and a reduction in risky behaviour such as adolescence crime and medicine use among mothers [33].

In Denmark, health visitors provide early parenting support to approx. 95% of all Danish families, mainly through home visits [31]. The Danish Health Authority recommends that health visitors have a family-focused approach to healthy child development and that they focus on mental health and the establishment of an early parent-infant relationship. Previous studies have revealed that Danish health visitors believed that they are highly motivated for their jobs, have a high level of self-efficacy, are knowledgeable and have good observation skills as far as assessing the parent-infant relationship is concerned [34]. However, the health visitors do not use specific methods for supporting the establishment of the early parent-infant relation [31]. Moreover, little is known about the effect of universal programmes in Danish health visitor services. Only one trial has been conducted, showing a positive effect of home visiting on breastfeeding duration [35].

### Rationale of the newborn behavioral observation (NBO) method

The NBO is a standardised method designed to assist health visitors in their facilitation of the parent-infant relationship through shared systematic observation of the infant with their parents. The method is based on the comprehensive Neonatal Behavioral Assessment Scale (NBAS) [36] and on clinical experience with infants and their families. The method consists of 18 neuro-behavioural observations of the infant when sleeping, being awake or crying [37]. So far, the NBO method has been tested in the UK among high-risk families [38] and in China and the US among depressed mothers and in a hospital setting [39–42]. The previous studies found that NBO may increases the quality of care related to parent-infant social interaction and reduces depressive symptoms among mothers. Even though health visitors in their daily practice in the UK and Norway use the NBO, the method has yet to be examined in a universal preventive programme with a strong design and a large study population.

## Methods

### Aim and hypothesis

The aim of this study is to examine the short-term and long-term effects on child and family outcomes of the implementation of a universal NBO method provided by health visitors to a general population of parents in a community setting. We hypothesize that early parental support facilitated by the standardised NBO will increase parental sensitivity, confidence and mood, and that it will reduced parental stress. Moreover, we hypothesize that it will lead to improved infant socio-emotional behaviour and cognitive development and improve the early parent-infant relationship during the first 18 months of the infant’s life compared with families who are receiving standard care from health visitors.

### Design

This is a cluster-randomised, community-based study with two parallel arms implemented in four Danish municipalities. Health visitors will conduct the trial, and the geographical districts they work in will constitute the clusters. The design includes a pre-test at baseline 1–2 weeks after birth and post-tests at 3, 9 and 18 months after birth. Both the intervention and the comparison groups will receive standard care by health visitors. In addition hereto, the intervention group of mothers and fathers will receive support as the standardised NBO method will be applied at home visits from 3 weeks to 3 months after birth.

### Setting and recruitment

The trial is conducted in four Danish municipalities. Recruitment of the primary study population of new families was initiated on 1 January 2017 and is expected to continue until 31 January 2018. Data collection was initiated on 15 January 2017 and is expected to continue until 30 September 2019. At the first home visit after birth, health visitors recruit all new parents to participate in the trial. During this first visit, families receive oral and written information about the project and about data collection through self-reported questionnaires and video recordings. Mothers and fathers give their informed consent before participating. Parents are not informed about the group to which they belong.

### Participants

The study population consists of all new families in the four participating municipalities during the study period. The study population of parents is expected to represent the following subgroups: First-time parents (40%), premature births (7%), twin or triplet births (2%), postpartum depressed mothers and fathers (7–14%) and parents with a cultural background other than Danish depending on geographical area (7–20%) [43, 44].

All mothers and biological fathers or maternal partners who are visited by a health visitor 1–2 weeks after birth are eligible for the study. As this is a community-based universal intervention with no expected side effects in which we seek to measure the effect in a natural population of new parents, we have no exclusion criteria except for parents who are undergoing treatment elsewhere or are unable to manage their own legal affairs and are therefore not visited by a health visitor.

### Intervention

The NBO will be used among new parents in the intervention group at the health visitor examination of the newborn during the home visit when the infant is 3 weeks old, and in any subsequent home visits until the age of 3 months [37]. Through shared observation with the parents, the aim of the standardised NBO method is to enhance the parents’ sensitivity to their infant’s unique capacities, to create an early understanding of the individual infant’s strengths and needs, and to help the parents to interpret cues and interact with their newborn infant [37]. During the 18 neuro-behavioural observations focusing on the infant’s autonomic, motor, social interactive behaviour and state, the health visitor actively involves the parents in the systematic observations and shares the translation of the newborn infant’s expressions and cues with the parents [36]. As a result of the shared observation and assessment, the health visitor can give individual guidance to the parents on relationship-building, sleeping and crying patterns, etc.

The new parents in the comparison group will receive care as usual by health visitors. Examination of the newborn infant is already a part of health visitors’ practice during home visits after birth [31]. Health visiting practice in both groups follow the guidelines of the Danish Health Authority except for the difference in practice relating to the examination of the newborn infant [31].

### Health visitors, implementation and adherence

All authorised health visitors employed in the four participating municipalities who conduct home visits with new families are eligible and will be included in the study. In June 2016 before project start, participating health visitors were randomised to either the intervention or the comparison group according to their geographical districts.

In October 2016, all health visitors working in districts randomised to the intervention group received a 2-day NBO training course by certified NBO trainers followed by a training phase in which the health visitors practiced and completed a minimum of five NBO recordings before achieving their NBO certification in December 2016. A number of strategies ensured that the delivery of the NBO was in line with the standardised version. Firstly, each health visitor received a manual on the NBO method for the training course, and all health visitors joined a 1-h team Skype session twice during the training phase. Secondly, teams of health visitors from the intervention group participated in a 1-day workshop in March 2017 and also in a 2-h supervision session every third month during the intervention period. The general aim of these strategies was to motivate health visitors and facilitate the delivery of the NBO method by giving the health visitors an opportunity to ask questions and achieve feedback.

A linkage group was established at the beginning of the project consisting of the health visitor leaders from each of the four participating municipalities, the NBO trainer and the scientific investigators. The aim of this group is to coordinate and facilitate communication and involvement between the fields of practice and researchers. The group meets every 4 months during the planning and intervention period.

The delivery and thus the degree of implementation of the NBO intervention is continually registered by health visitors in the intervention group as they fill in a checklist during each home visit performed 3 weeks after birth to monitor which parts of the NBO was delivered during the visit. Additionally, data collection among the health visitors in both the intervention and comparison group aims at following any change in the health visitors’ knowledge, observation skills, intention and self-efficacy in their work with early parent-infant interactions.

### Data collection and study population

Data collection will continue among the participating parents until the child reaches 18 months of age. The data will consist of self-reported data and data coded from assessment of video interactions. Self-reported data from parents will be obtained at four time points via separate questionnaires to the mothers and fathers/partners at baseline 1–2 weeks postpartum and at follow-up at 3, 9 and 18 months postpartum. Questionnaires are collected via a web-based system with personal log in. Two reminders are sent by e-mail and SMS. Supplemental printed questionnaires are available for participants who do not have a computer or lack access to the internet. No monetary incentives are used to motivate participation. Lottery prizes representing a value of DKK 2000 will be drawn monthly from the pool of participants who complete the questionnaire.

In both the intervention group and the comparison group, a subgroup of 90 first-time mothers is selected consecutively among the responders for video recordings, which will be made about half way through the project period. The specified criteria for the subgroup are a low parenting confidence score, < 40 on the Karitane Parenting Confidence Scale (KPCS) [45], symptoms of depression (> 20 on the Major Depression Inventory (MDI) [46, 47]) or premature birth (< 37 gestational weeks). The aim of the video recording is to collect observational data to assess the quality of the mother-infant interaction. Data from the video recordings will be obtained at 4 months postpartum, adjusting for the age of premature infants [48]. The subgroup of mothers will be instructed to be with and interact with their infant as normally [48] while a 3-min video is recorded by a health visitor. Video coding will be performed by certified blinded CARE-Index raters; and to ensure inter-rater reliability, a random blinded sample will be coded and tested initially.

To minimise any bias introduced by data collection, data are collected in the same way and with the same timing in the intervention and comparison groups. Supplemental register data will be collected among non-participants and non-responders to facilitate assessment of the drop-out rate and its impact on the results.

### Outcome measures

The outcomes of the intervention are measured within three main domains: Parental status and development over time in parental confidence, mood, and stress; infant status and development over time in social, emotional and cognitive development; and, finally, interaction between parents and infant focusing on mother and infant interaction. The main outcomes are measured by validated scales and the Infant Care Index, a validated video-based observation system [48]. The main outcomes appear from Table 1.

**Table 1:** Outcome variables for the primary study population and timing of data-collection

Separate self-reported questionnaire from the mother and father	2 weeks	3 months	9 months	18 months
Outcomes – parents				
Karitane Parenting Confidence Scale (KPCS)	x	x	x	
Major Depression Inventory (MDI)	x	x	x	x
Outcomes - infants				
Ages and Stages Questionnaire: Social-Emotional (ASQ:SE)	x	x	x	x
The Strengths and Difficulties Questionnaire (SDQ)				x
Ages and Stages Questionnaire (ASQ)			x	
Family relationship				
Mother and Baby Interaction Scale (MABISC)	x	x	x	x
Video observation mother-infant interaction, CARE-Index		x		

### Primary outcomes

*The Karitane Parenting Confidence Scale (KPCS)* by Črnčec et al. (2008) [45]. The KPCS consists of a 15-item questionnaire measuring parenting confidence in parents to infants aged from 0 to 12 months of age. The KPCS is rated on a four-point scale. The KPCS has not been validated in a Danish setting, but in an Australian context where it showed a good level of sensitivity (86%) and specificity (88%) [45]. The wording of the questions can be found in Črnčec et al. (2008) [45].

*The Ages and Stages Questionnaire: social-emotional (ASQ:SE)* by Squires et al. (1997) [49] consists of a questionnaire with 19–33 items measuring social-emotional problems and competencies in children regarding self-regulation, compliance, adaptive functioning, autonomy, affect, social communication and parent-children interaction in children aged from 1 month to 5 years. The ASQ:SE is rated by parents on a three-point scale; it includes a box where parents may report if the behaviour of their infant/child is a concern for them. The ASQ:SE has not been validated in a Danish context but internationally, showing a moderate to good sensitivity of 71–85% and an excellent specificity of 90–98% [49].The wording of the questions can be found in Squires et al. (1997).

*Video of mother-infant interaction coded by the Infant CARE-Index* by Crittenden (2006) consists of a video recording to assess the quality of mother-infant interaction (dyadic synchrony), the mother’s sensitivity and the infant’s cooperation in the interaction. The tree variables dyadic synchrony, maternal sensitivity and infant cooperation will be rated on a 0–14 point scale [48]. The 3-min videos will be collected 4 months after birth and coded by certified coders according to the Infant CARE-Index method [48]. To ensure the inter-rater reliability, the Cronbach’s alpha coefficient test of a consecutive blinded sample of the first ten collected video recordings will be analysed.

### Secondary outcomes

*The Major Depression Inventory* (MDI) by Olsen et al. (2003) [47] consists of a 10-item questionnaire measuring parental symptoms of depression on a six-point scale. The wording of the questions can be found in Olsen et al. (2003).

*The Mother and Baby Interaction Scale* (MABIC) by Hackney (1996) [50] consists of a 10-item questionnaire measuring the mother-infant relationship on a five-point Likert scale. The wording of the questions can be found in Hackney (1996).

*The duration of exclusive breastfeeding and any breastfeeding* according to the definition of the WHO [51] is measured in the weeks following birth.

*The Ages and Stages Questionnaire: (ASQ-3)* by Squires et al. (2016) [52] is a 21-item questionnaire measuring communication, gross motor skills, fine motor skills, problem solving and personal-social competencies in children aged from 1 month to 5 years. The ASQ is rated by parents on a three-point scale [49]. The wording of the questions can be found in Squires et al. (2016).

*The Strengths and Difficulties Questionnaire (SDQ)* by Goodman (2001) [53] consists of a 25-item questionnaire and measures five domains: hyperactivity, peer problems, conduct problems, emotional symptoms and pro-social behaviours rated by parents on a three-point scale. The wording of the questions can be found in Goodman (2001).

### Background information and mother, father and infant characteristics

Data collected on the background characteristics of mothers, fathers and infants include: parental age, gender, marital status, educational level, employment status, smoking habits, height and weight, well-being WHO-Five [54] and Coupes Satisfaction Index [55]. Concerning the infant: place of birth, gestational age, and questions about infant development. Supplemental data collected from the services received from health visitors and medical services include infant contacts to GP, the emergency room and hospitals within the first year of life.

Background socioeconomic information on eligible parents who do not enrol in the study is available from register data. This allows for comparison of participants in the randomised groups with the overall population, and for examination of the heterogeneity of programme effects across family types.

### Data collection health visitors

Data from health visitors are collected at three time points: Before the NBO training course in October 2016 (only the health visitors of the intervention group), before the intervention period in December 2016 and after the intervention period in February 2018. Self-reported questionnaires are obtained via printed questionnaires; and measures include: *Observation skills:* The ability to observe the quality of parent-infant interactions was measured by asking the health visitors to observe and assess four video-recordings on mother-infant interactions regarding the mother’s sensitivity and the mother-infant dyadic synchrony [48]. *Knowledge about early relationship:* Infant’s signs of stress, emotional self-regulation and how to comfort an infant were measured by asking health visitors about their general knowledge in a four-item questionnaire. *Intention:* Valuation of personal goals for working with early relationships was measured by one question and rated on a Likert scale. *Self-efficacy:* Confidence in the ability to work with early relationships and skills in supporting and guiding and capacity to cope with insecure first-time mothers to establish a healthy relationship with their newborn infants were measured using a 12-item questionnaire. *Background information and characteristics of the participating health visitors:* data on age, work experience and additional education and training.

### Sample size

Ongoing research shows that 25% of new mothers have a risk score < 40 on the KPCS [28]. Building the power calculation on an assumed loss to follow-up of 15%, a significance level of 5% and a power of 80%, calculations showed a need for 934 participating mothers to be able to identify a statistically significant difference decrease of 8% in reporting low confidence in understanding their infants. Taking an average of the relatively large cluster size into account (1 + (cluster size-1)*ICC), the desired number of participating mothers will be 934 X (1 + (200–1)*0.01) = 2793 mothers [56]. The included intraclass correlation coefficient (ICC) in the calculation is relatively low because the self-reported outcome is collected individually, and we do not expect mothers within clusters to be more alike than mothers across clusters. Moreover, earlier findings in similar research have shown a low cluster effect [35]. With the current birth rate in the participating municipalities, the number will require an inclusion and project period of approximately 12 months with follow-up at 3, 9 and 18 months.

### Randomisation

The cluster randomisation was carried out in June 2016 after receiving the declaration about participation from the municipalities and before the health visitors’ enrolment in the NBO training course. The already established geographical districts within the four participating municipalities where health visitors provide home visits to new families were regarded as clusters (*n* = 17) to avoid a spill-over effect between health visitors and families. Thus, health visitors were allocated to an intervention or comparison group according to their geographical districts, whereas the participating families were allocated according to their health visitor.

Prior investigation of the clusters alias the districts revealed that all districts represented a variation in social status among inhabitants and considerable differences in birth rates; in 2015, from 50 to 200 births. The birth rates were associated with the number of health visitors employed in each district. Because of the relatively small number of clusters, we therefore decided to use a restricted randomisation procedure to achieve balance between the two study arms [57]. The criterion for the restricted randomisation was a geographical balance with all participating municipalities represented by both intervention and comparison districts. Moreover, we attempted to obtain numerical balance with the expected number of births between the intervention and the comparison group. In each municipality, the districts were divided into two groups. For each municipality, the districts were allocated randomly to one of the two treatment groups ensuring that both treatments appeared in each municipality and that the treatment groups had approximately the same expected number of births as the comparison groups. An independent data manager handled the entire randomisation procedure.

### Statistical methods

Our data analyses will be in accordance with the CONSORT guidelines [58]. Intention to treat analysis will be used to detect the effect of the community-based intervention where participation may vary in the study population of parents. Following the intention to treat principle, mothers and fathers will remain in the group defined by the cluster randomisation. A baseline comparison of pre-defined background variables in the two groups will include unpaired t-tests and Fisher’s exact test. When testing the NBO with treatment as usual, the mean scores and any change in the primary variables across time in the intervention group from pre-test (at 2 weeks) to post-test at3, 9 and 18 months will be compared to mean scores and changes in the comparison group. Supplemental analyses will be performed to identify an intervention effect for subgroups and marginal groups of parents according to parity, preterm delivery and depression symptoms. Analyses will include multiple regression analysis to adjust for pre-randomisation variables such as parents’ age, marital status and educational background. Due to the small number of clusters, the analysis will include adjustment for within-cluster correlation. As we examine a relatively large number of outcomes, we will assess the robustness of our findings by applying methods suitable for multiple hypotheses testing. Finally, we will perform a drop-out analysis and examine the comparability of intervention and comparison group in the trial with respect to differences in received health visitor services.

### Harms

The NBO method developed by Brazelton builds on clinical studies of newborn infants and assumes that they have a range set of skills and social abilities that allow them to communicate with their surroundings [38, 59]. As the method takes as its starting point the newborn infant’s condition and cooperation with the parents, we expect no side effects or risks associated with the intervention for the participating infants or parents.

## Discussion

The protocol describes a cluster-randomised community-based trial on a universal parenting support programme (NBO) currently being rolled out in Denmark. The programme efficiency has not yet been studied in a general population in Denmark or internationally.

The NBO method is a clinically relevant alternative to the non-standardised observation of the newborn infant in health visitor practice. By comparing the effect of the NBO method with usual practice, the study contributes to building a more evidence-based practice for health visitors.

The study is community-based, representing a diverse population of new parents. By including all new parents who receive health visitor services in the participating municipalities, we can measure the effect of the NBO method in a universal context. The study population includes subgroups of new parents. Because of their different characteristics, they may be affected in different ways by having a new-born infant and by the support they receive. This may have implications for the interpretation of any study findings.

The randomisation procedure reflects a trade-off between the theoretically optimal procedure and what was possible in practice in a community trial. The already established geographical districts in the participating municipalities were used as clusters to avoid contamination between newly educated health visitors in the intervention group and health visitors who had not received such training in the comparison group. Restricted randomisation was used to minimise imbalances between treatment arms because the heterogeneous characteristics of the districts regarding birth rates were known prior to randomisation. The reduced degree of randomness introduced by clusters and restrictions may increase the risk of selection bias. However, all districts/clusters were enrolled prior to randomisation, had the same chance of receiving the treatment, and learned at the same time of their allocation. Additionally, achieving balanced study arms in our cluster-randomised trial may contribute to the interpretation of study findings, whereas unbalanced study arms may create methodological problems with inference of certain conclusions [57].

The intention of this community-based trial is to test the NBO method as a preventive intervention for development of early healthy parent-infant interaction in a diverse population of new parents. A monitoring process in clinical practice follows the implementation and dissemination of the method. The outcome is tested on a broad range of health outcomes related to the mother, the father and the infant, as establishing an early relationship between parents and their infant is a common health issue influencing a broad range of subsequent health-related problems.

Results will be prepared for publication in international peer reviewed journals in the fields of public health, developmental psychology and nursing science.

## Abbreviations

ASQ: Ages & Stages Questionnaires
ASQ:SE: Ages & Stages Questionnaires, Social-Emotional
KPCS: Karitane Parenting Confidence Scale
MDI: Major Depression Scale
NBO: New-born Behavioral Observation
SDQ: Strengths and Difficulties Questionnaire
SS: Parental Stress Scale
